# Synthesis, description, and application of novel corrosion inhibitors for CS AISI1095 in 1.0 M HCl based on benzoquinoline derivatives

**DOI:** 10.1038/s41598-023-39714-1

**Published:** 2023-08-23

**Authors:** Ali G. Sayed, Ashraf M. Ashmawy, Walid E. Elgammal, Saber M. Hassan, M. A. Deyab

**Affiliations:** 1 Chemistry Department, Faculty of Science (Boys), Al-Azhar University, Nasr City, Cairo 11884 Egypt; 2 Egyptian Petroleum Research Institute (EPRI), Nasr City, Cairo Egypt

**Keywords:** Chemistry, Electrochemistry

## Abstract

This study aims to synthesize and evaluate the corrosion inhibition properties of three newly prepared organic compounds based on benzo[h]quinoline hydrazone derivatives. The compounds structure were characterised using FTIR, 1H-NMR, 13C-NMR and Mass spectroscopy. Electrochemical methods, including Potentiodynamic Polarization (PP), Electrochemical Frequency Modulation (EFM), and Electrochemical Impedance Spectroscopy (EIS) were employed to evaluate the compounds as corrosion inhibitors in HCl (1.0 M) for carbon steel (CS). Additionally, surface examination techniques such as scanning electron microscopy (SEM) and energy-dispersive X-ray spectroscopy (EDX) were used to investigate the surface morphology and elemental composition of the CS before and after exposure to the synthesized compounds. The electrochemical measurements showed that compound VII achieved corrosion inhibition efficiency. SEM and EDX analysis further confirmed the creation of a passive film on the CS surface. These findings demonstrated the potential of benzo[h]quinoline hydrazone derivatives as effective organic corrosion inhibitors for CS in aggressive solution.

## Introduction

The degradation of metals or alloys that occurs naturally and irreversibly because of chemical or electrochemical reactions with the environment is known as corrosion^1^. It has a significant negative influence on the industrial sector since it weakens important industrial resources like iron and its alloy which are considered the backbone of modern industry, because of their inexpensive cost, ease of manufacture, high tensile strength, high heat stability, and outstanding mechanical qualities^2^. According to NACE study, corrosion is estimated to cost the world annually about US $ 2.5 trillion, or 3.4% of GDP Gross domestic product in 2013^3,4^. There are numerous potential methods for controlling corrosion such as organic corrosion inhibitors, protective coating, alloying, and functionalized carbon dots. These techniques are created and applied based on the environment, the type of metal, and the electrolyte^5^. Organic corrosion inhibitors are one of the finest choices because of their relatively low price, high corrosion inhibition efficiency, and environmentally friendly behaviour^6^. In general, literatures reported that heterocyclic compounds with conjugated double bonds and polar groups, such as sulphur, nitrogen, oxygen are effective inhibitors of iron corrosion^7–9^. These substances work by binding the metal surface via chemisorption, physisorption, complexation, or precipitation to block oxygen from reaching the cathode, block hydrogen from diffusing from the cathode, or block metal dissolution (anodic inhibitors)^10^. Recent studies focused on finding new corrosion inhibitors that satisfy the necessary requirements from the technical standpoint, with the most emphasis being placed on inhibitors that are non-toxic or environmentally safe^11,12^.

Although quinoline based compounds are well kwon to have a variety of biological activities, and their potential as antibacterial, anti-Alzheimer, antifungal, anticancer, intra-malarial, and anti-HIV (human immunodeficiency virus) agents has received considerable attention^13–17^. It makes sense that quinoline and benzoquinoline derivatives also satisfy all criteria for effective corrosion inhibitors. Their corrosion inhibition activities were reported in some previous studies, and they performed well, especially in the acidic medium^18–20^.

The primary goal of this study was the synthesis of three novel benzo[h]quinoline hydrazone derivatives, which highlights its novelty. A nitrogenous tricyclic planar heterocyclic compound known as benzo[h]quinoline has been previously described as a potent corrosion inhibitor^21,22^. We enhanced this structure by adding various groups and Schiff base hydrazones to obtain derivatives of nicotinoyl hydrazone, benzoyl hydrazone, and acetyl hydrazone. Since we increased the aromaticity, electron density, and active sites, this improvement appears to have the potential to enhance the effectiveness of the synthesised compounds ability to inhibit corrosion.

Our study extended to characterize the structure of the novel compounds using FTIR, ^1^HNMR, ^13^CNMR and mass spectroscopy. Then, their performance as corrosion resistance for CS evaluated serval techniques including electrochemical and surface examination techniques. We evaluated the effectiveness of all these corrosion inhibitors in HCl (1.0 M) since the mineral acid solutions are widely used in industrial applications especially the hydrochloric acid which is most effective and economical to be used in; disinfection, oil well cleaning, chemical treatments, closed circuits, and as pickling agents^23–25^.

Table 1 compares one of the inhibitors we investigated with those from the literature that are comparable in terms of chemistry and active function groups^26–28^.

**Table 1:** Comparison of the Inhibition Efficiency of our investigated inhibitors with other inhibitors from the literature.

Inhibitor	Medium	Metal	Concentration	IE%, according To PP technique	References
(E)-2-(1-triazylidineethyl)pyridine	1 M HCl	Mild steel	10^−3^ M	77.7	Binsi et al.^26^
Quinolinyl thiopropano hydrazone derivative	1 M HCl	Mild steel	11.08 × 10^−4^ M	88.9	Saliyan et al.^27^
Denzoyl benzaldehyde hydrazone derivative	1 M HNO_3_	Copper	1.6 × 10^−5^ M	80.8	El-Shafel et al.^28^
N'((2chlorobenzo[h]quinolin3yl)methylene) benzohydrazide	1 M HCl	AISI1095 Carbon Steel	500 PPM	90.33	This study

## Experimental section

### Materials

The composition (weight %) of the applied carbon steel sheets is 1.86C, 2.51O, 0.74 Al, 0.24P, 0.23S, 1.09Mn and 93.33Fe. The electrode used in the electrochemical investigations has a total surface area of one cm^2^.

### Chemicals

All starting materials, solvents, and reagents, which were used for the synthesis of the inhibitors were gained from commercial suppliers directly and used as received unless otherwise shown. HCl (1.0 M) was prepared by freshly dilution of analytical grade 37% HCl (Sp. Gr. 1.2 g/mL) using distilled water. A solution that is acidic was used to dissolve inhibitors at concentrations ranging from 100 to 500 ppm.

### Characterization of the inhibitors

The fusion point of the synthesized molecules FTIR was specified via SMP50 Digital Melting Point APP at 120/230 V “Bibby Scientific, Staffordshire, UK”. The IR spectrum was recorded at potassium bromide tablets (4000–400 cm^−1^) using a Nicolet “iS10FT-IR Spectrometer and Thermos-Fisher Scientific Resolution16”. ^1^HNMR and ^13^CNMR) spectra were taped at 300 MHz on a Varian “VX-300 NMR spectrometer” and Bruker EX-400 MHz (^13^C: 100 MHz) spectrometer) (Bruker, USA) respectively, in D_2_O (dimethyl sulfoxide was used as an external standard, [δ(^1^H) = 2.50 and 3.31 ppm, δ (^13^C) = 39.52 ppm]. The chemical shifts are provided in ppm relating to tetramethyl silane (TMS) at 0.00 ppm. At the Regional Centre for Mycology and Biotechnology, Al-Azhar University, Egypt, mass spectra were collected using a “Thermo Scientific Gcms) Model—Japan”. In addition, the results of the elemental analysis were established and were correct to 0.4%.

### Synthesis of inhibitors

#### Synthesis of N-acetyl-1-naphthylamine. (I)

The traditional method for the preparation of N-(naphthalen-1-yl) acetamide(I). Compound (I) was synthesised as it was described in the publication^29^ with slight changes. A 100 round-bottom flask (RBF), equipped with a reflux condenser system, was charged with a mixture of (α-naphthylamine (0.1 mol) and acetic anhydride (0.1 mol, 99%) in alcohol (CH_3_OH, 25 mL). After adding a glacial acetic acid in a catalytic amount, the resultant mixture was refluxed in a water bath for one hour. At the end of the period, the crude was poured directly onto crushed ice, after cooling to room temperature at the end of the interval. The resulting filtered solid was washed with water and crystallized from methyl alcohol. (White solid, 93% yield, melting point 156–158 °C.) (Lit.: 158–160 °C).

#### Synthesis of 2-chlorobenzo[h]quinoline-3-carbaldehyde. (II)^30^

At 0 °C, 10 mmol of compound (I) was progressively transferred to a solution of Vilsmeier-Haack reagent (POCl_3_ (70 mmol) and DMF (30 mmol) in a 100 ml flask equipped with a mechanical stirrer (1 h) and enclosed by an ice–salt bath. Then, the cooling bath was removed, and the mixture was heated at 80 °C for 15 h. The reaction mixture was then placed onto the ice and allowed to sit for an overnight period before being neutralized with solid NaHCO_3_. The produced solid was recrystallized from a petroleum ether/ethyl acetate mixture (1:1) after filtration and washing to give the desired product. (Yellow needles, 72% yield, 208–210 °C melting point).

#### General method for the synthesis of N-(benzo[h]quinoline-3 ylmethylene) hydrazide derivatives. (VI-VIII)

Isonicotinohydrazide (III, 1 mmol), benzohydrazide (IV, 1 mmol), or acetohydrazide (V, 1 mmol) was added to an equivalent amount of 2-chlorobenzo[h] quinoline-3-carbaldehyde (II, 1 mmol) in hot 1,4-dioxane (25 ml), followed by a catalytic quantity of glacial acetic acid, was added. For four hours, the reaction mixture was refluxed. After completing the reaction, the solid appeared upon cooling, was filtered, washed with cold dioxane, dried, and recrystallized with a proper solvent to give the target compounds (VI–VIII).

##### N'((2chlorobenzo[h]quinolin3yl)methylene)isonicotinohydrazide(VI)

**Figure 1:**
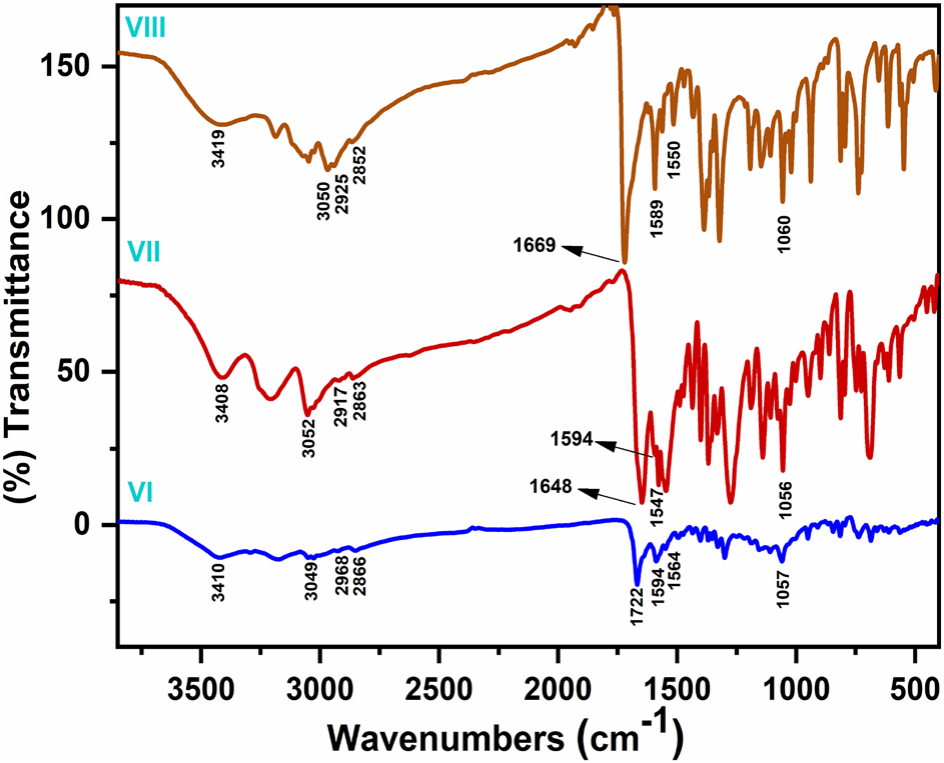
FT-IR of corrosion inhibitors VI, VII, and VIII.

**Figure 2:**
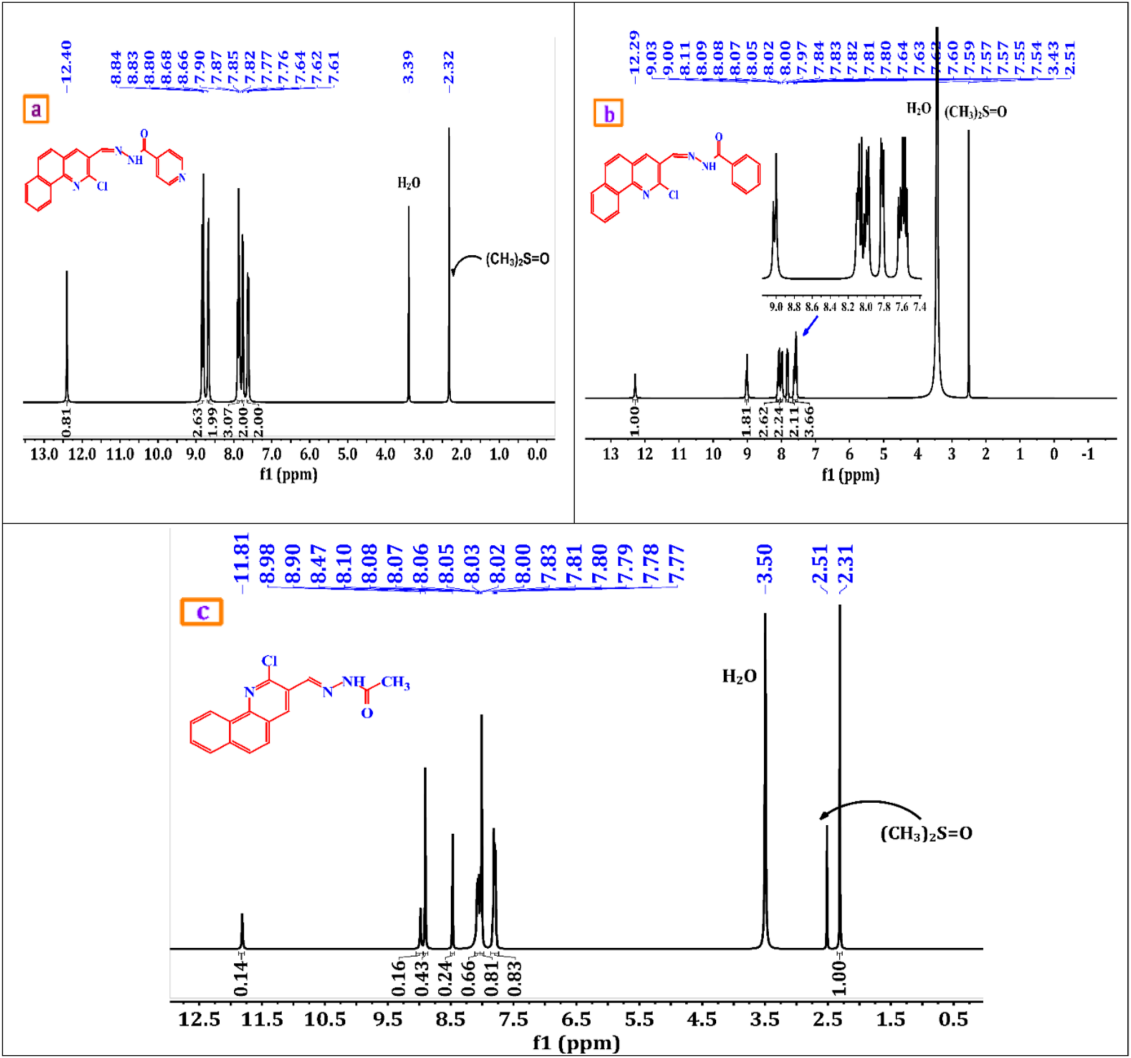
(**a**–**c**) ^1^H-NMR of corrosion inhibitors VI, VII, and VIII.

**Figure 3:**
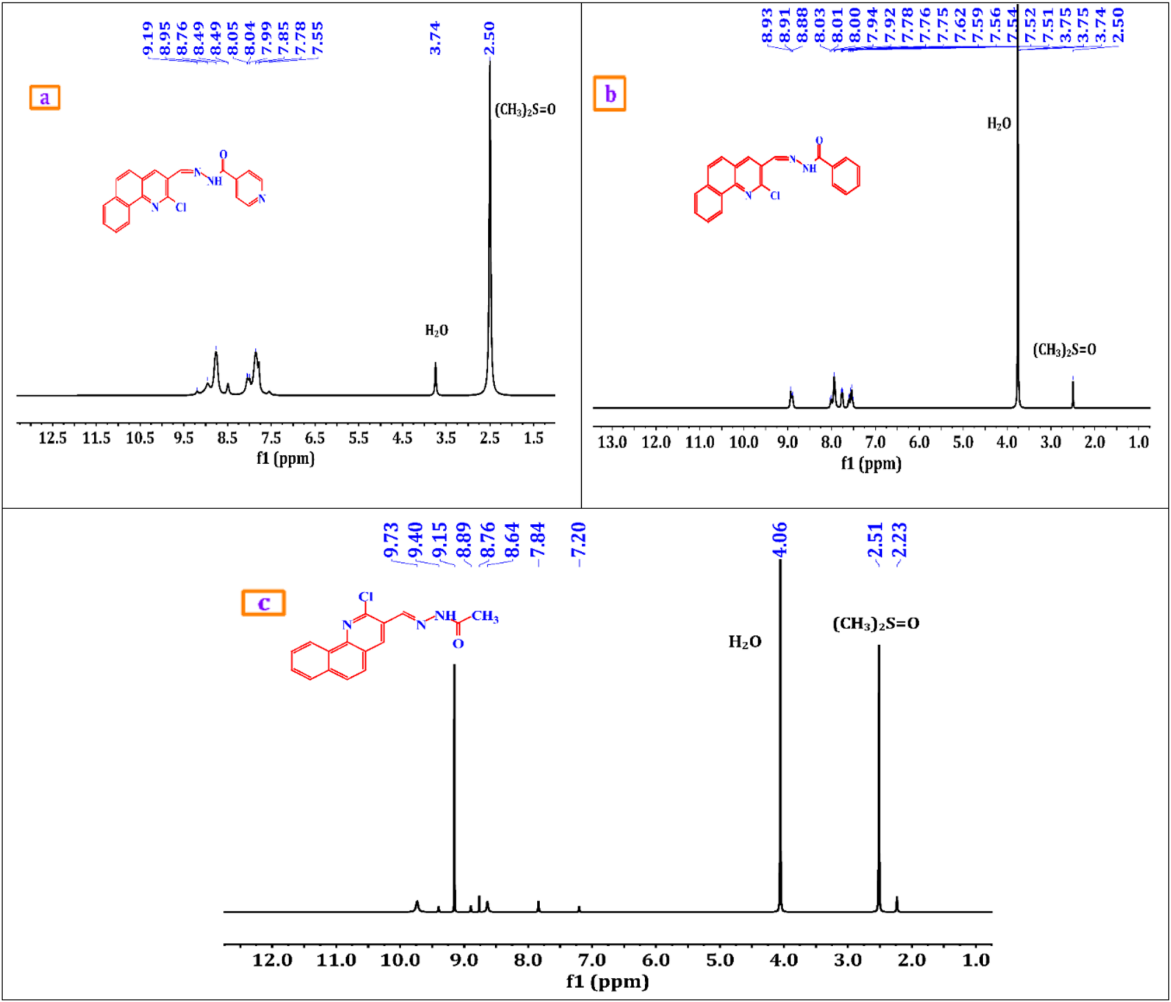
(**a**–**c**) ^1^H-NMR/D_2_O of corrosion inhibitors VI, VII, and VIII.

**Figure 4:**
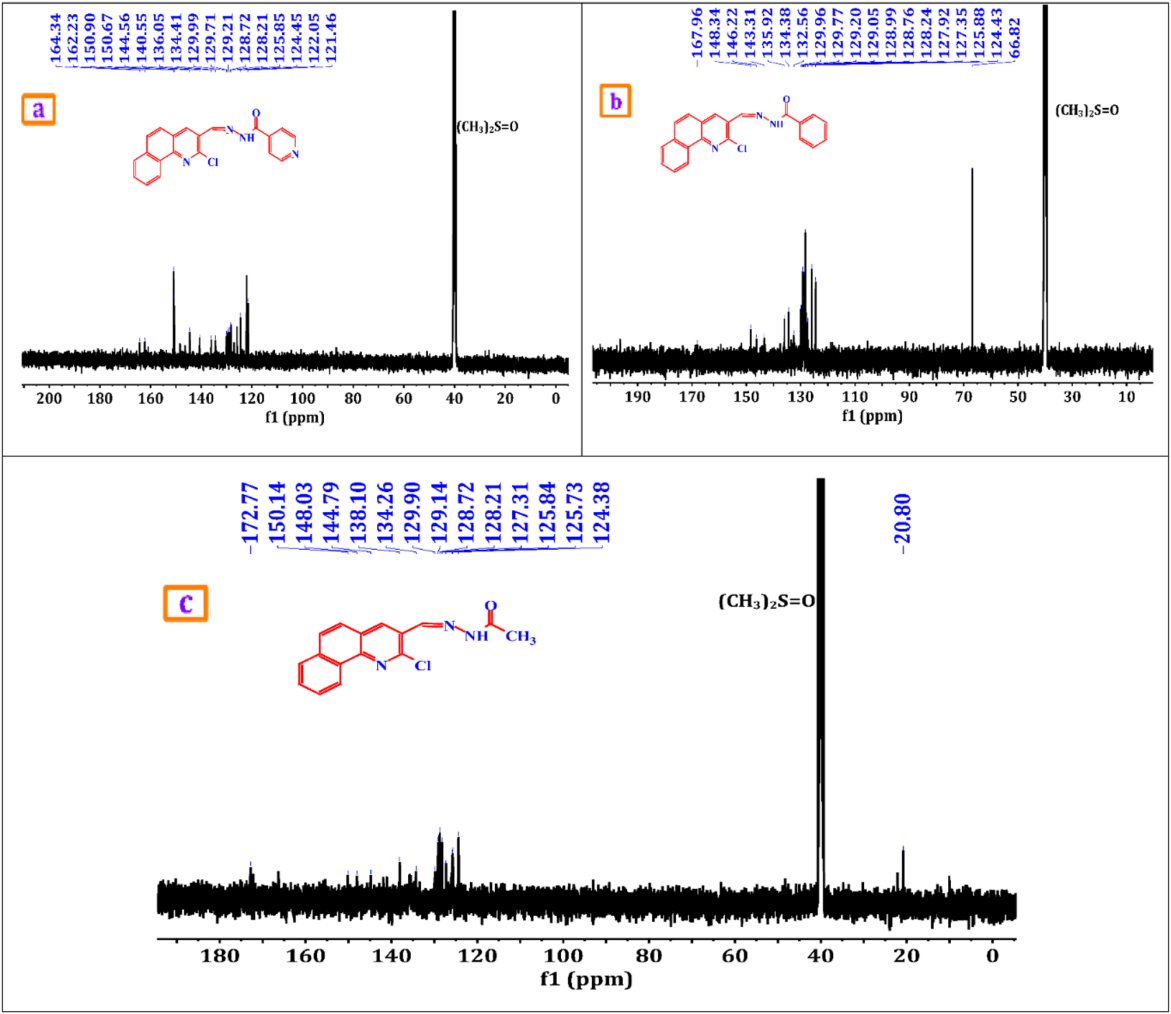
(**a**–**c**) ^13^C-NMR of corrosion inhibitors VI, VII, and VIII.

**Figure 5:**
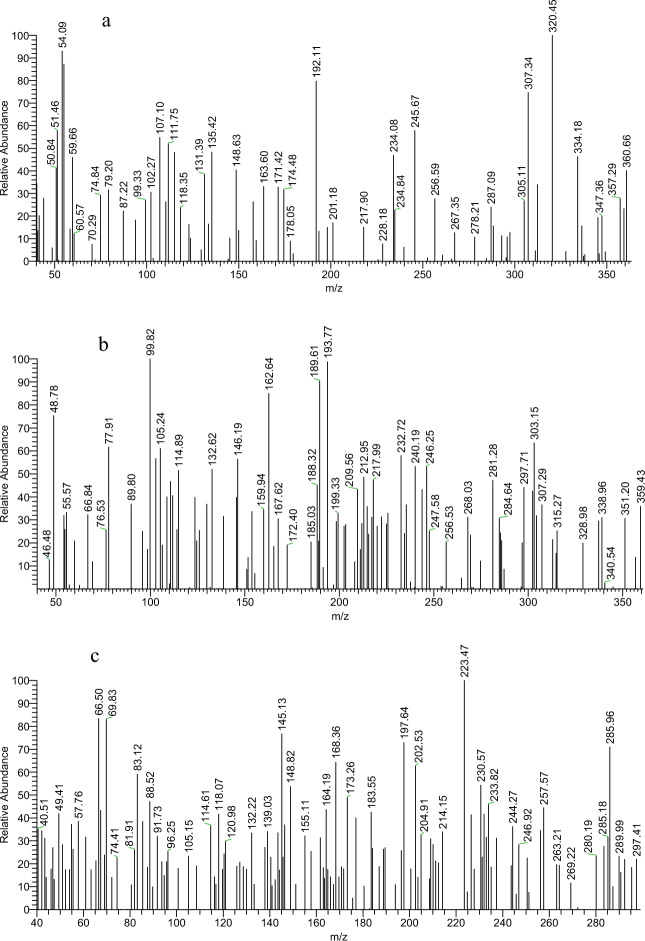
(**a**–**c**) Mass spectrum of corrosion inhibitors VI, VII, and VIII.

**Figure 6:**
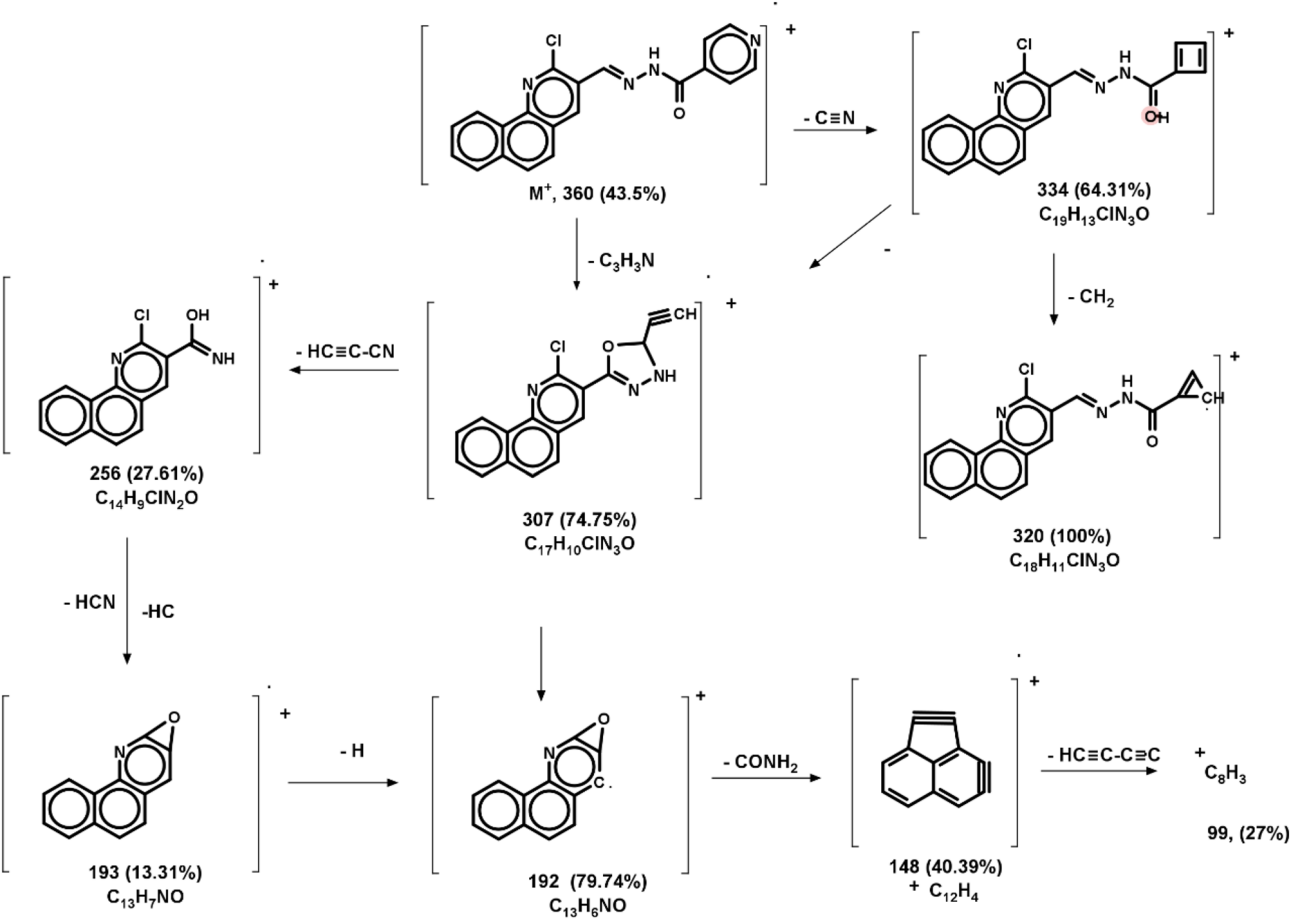
Mass fragmentation of compound VI.

A brownish-yellow powdered, DMF: MeOH (9:1), yield 75%, melting point 250–252 °C; FT-IR (KBr-tablet) (*υ*_max_/cm^−1^) (Fig. 1): 3419 cm^−1^ (NH), 3050 cm^−1^ (CH-aromatic), 2925, 2852 cm^−1^ (CH-aliphatic) (asymmetric and symmetric), 1669 cm^−1^ (C=O), 1589 cm^−1^ (C=N), 1550 cm^−1^ (C=C) and 1060 cm^−1^ C–Cl. ^1^H-NMR (DMSO-d_6_, 300 MHz); (δ, ppm) (Figs. 2a, 3a): 12.57 (s,1H, NH, exchangeable by D_2_O), 9.02 (s. 1H, H_4_-quinoline), 8.97 (s, 1H, CH=N, azomethine), 8.84,7.94 (dd, *J* = 5.1 Hz*,* 4H, isonicotonyl), 8.11–7.98 (m, 3H, H_**5,7,10**_-benzoquinoline), 7.90–7.73 (m, 3H, H_**6,8,9**_-benzoquinoline). ^13^C-NMR (DMSO-d_6_,101 MHz); (δ, ppm) (Fig. 4a): 164.34 (isonicotonyl C=O), 162.23 (quinoline C2), 150.90 (quinoline C10b), 150.67 (isonicotonyl C–N), 144.56 (azomethine CH=N), 140.55 (isonicotonyl C=C), 136.05 (quinoline C4), 134.41 (quinoline C6a), 129.99 (quinoline C10a), 129.71 (quinoline C5), 129.21 (Benzoquinoline C7), 128.72 (Benzoquinoline C8, C9), 128.21 (quinoline C4a), 125.85 (quinoline C6), 124.45 (Benzoquinoline C10), 122.05 (quinoline C3), 121.46 (isonicotonyl C=C). Further, the structure of **(VI)** was also confirmed by recording its mass spectrum (Fig. 5a), and as shown in mass fragmentation (Fig. 6), which manifested a molecular ion peak at m/z = 360.08 [%]: [M + , (40.07%)], assigned to the molecular ion, which losses, C_3_H_3_N, HC≡C–CN, HCN & HCl, H, CONH_2_, and HC≡C–C≡C^·^ respectively to give fragment peaks at (307; 74.75%), (256; 27.61%), (193; 13.31%), (192; 79.74%), (148; 40.39%), and (99; 27%) respectively. The base peak appeared at (320; 100%) due to the loss of C≡N and CH_2_ from the molecular ion peak.

##### N′-((2-chlorobenzo[h]quinolin-3-yl)methylene)benzohydrazide(VII)

**Figure 7:**
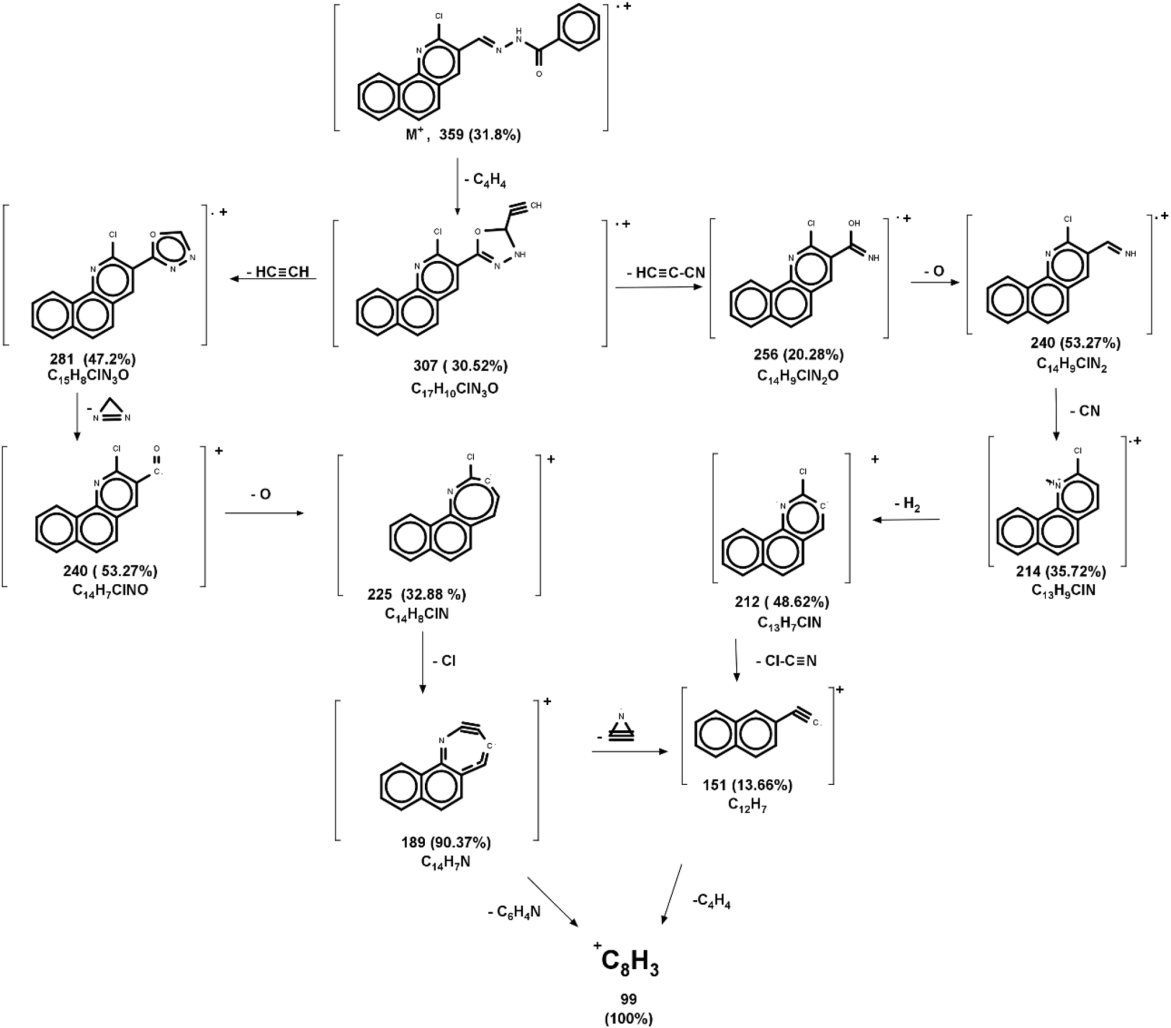
Mass fragmentation of compound VII.

A brownish-yellow powdered, DMF: MeOH (9:1), yield 82%, melting point 224–226 °C; FTIR (KBr-tablet) (*υ*_max_/cm^−1^) (Fig. 1): 3408 cm^−1^ (NH), 3053 cm^−1^ (CH-aromatic), 2917, 2863 cm^−1^ (CH-aliphatic) (asymmetric and symmetric),1648 cm^−1^ (C=O), 1594 cm^−1^ (C=N), 1547 cm^−1^ (C=C) and 1056 cm^−1^ (C–Cl). ^1^H-NMR (Fig. 2b, 3b): 12.29 (s,1H, NH, exchangeable by D_2_O), 9.03 (s. 1H, H_4_-quinoline), 9.00 (s, 1H, CH=N, azomethine), 8.11–7.98 (m, 11H, Ar–H).^13^C-NMR (Fig. 4b): 167.96 (benzohydrazide C=O), 148.34 (quinoline C2), 146.22 (quinoline C10b), 143.31 (azomethine CH=N), 135.92 (quinoline C4), 134.38 (Benzoquinoline C6a), 132.56 (ArC), 129.96 (ArC), 129.77 (Benzoquinoline C10a), 129.20 (ArC), 129.05 (quinoline C5), 128.99 (ArC), 128.76 (Benzoquinoline C7), 128.24 (Benzoquinoline C8, C9), 127.92 (quinoline C4a), 127.35 (quinoline C6), 125.88 (Benzoquinoline C10), 124.43 (quinoline C3). Further support the compound's molecular formula, a mass spectrometry examination of the compound's molecular formula (Fig. 5b), and as clear in mass fragmentation (Fig. 7), which displays the peak at m/z = 359.08 [%]: [M^+^, (35.90%)], related to the molecular ion, which losses C_4_H_4_, HC≡CH, CN_2_H, O, Cl, and C_6_H_4_N respectively, to give fragment peaks at (307; 30.52%), (281; 47.20%), (240; 53.27%), (225; 32.88%), (189; 90.37%) and Base peak at (99; 100%) respectively. Also, molecular ion peak losses C_4_H_4_, HC≡C–CN, O, CN, H_2_, Cl–C≡N, and C_4_H_4_ respectively to give peaks at (307; 30.52%), (256; 20.28%), (240; 53.27%), (214; 35.72%), (212; 48.62%), (151; 13.66%) and base peak at (99; 100%) respectively.

##### N′-((2-chlorobenzo[h]quinolin-3-yl)methylene)acetohydrazide. (VIII)

**Figure 8:**
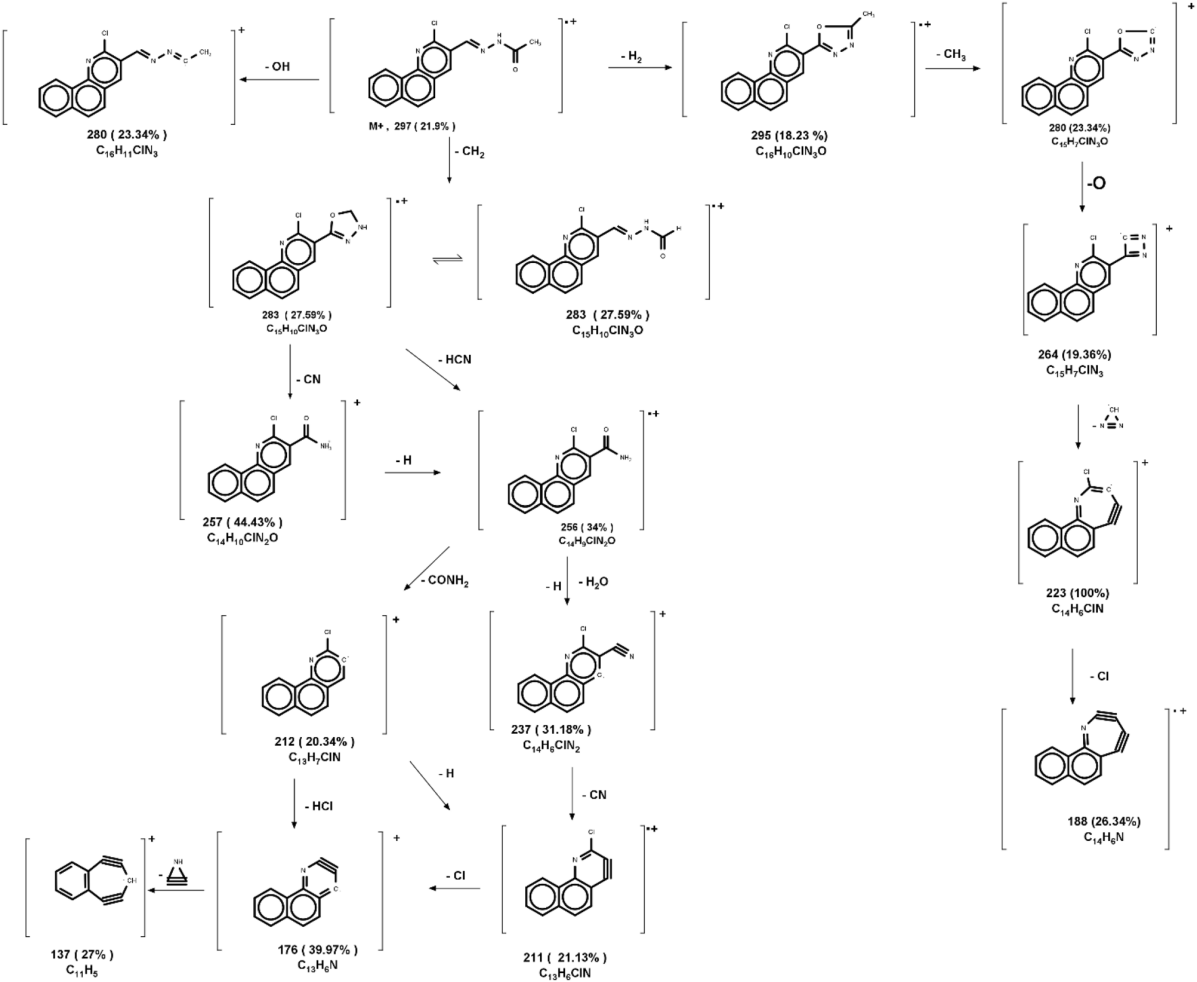
Mass fragmentation of compound VII.

A yellow powdered, DMF: MeOH (9:1), yield 68%, melting point 306–308 °C; FTIR (KBr-tablet) (*υ*_max_/cm^−1^) (Fig. 1),: 3410 cm^−1^ (NH), 3049 cm^−1^ (CH-aromatic), 2968, 2866 cm^−1^ CH-aliphatic) (Asymmetric and symmetric),1722 cm^−1^ (C=O), 1594 cm^−1^ (C=N), 1564 cm^−1^ (C=C), and 1057 cm^−1^ C–Cl.). ^1^H-NMR (Figs. 2c, 3c): 11.81 (s,1H, NH, exchangeable by D_2_O), 8.98 (s. 1H, H_4_-quinoline), 8.90 (s, 1H, CH=N, azomethine), 8.47–7.77 (m, 6H, Ar–H) and 2.31 (s,3H, CH_3_C=O). ^13^C-NMR (Fig. 4c): 172.77 (acetohydrazide C=O), 150.14 (quinoline C2), 148.03 (quinoline C10b), 144.79 (azomethine CH=N), 138.10 (quinoline C4), 134.26 (Benzoquinoline C6a), 129.90 (Benzoquinoline C10a), 129.14 (quinoline C5), 128.72 (Benzoquinoline C7), 128.21 (Benzoquinoline C8, C9), 127.31 (quinoline C4a), 125.84 (quinoline C6), 125.73 (Benzoquinoline C10), 124.38 (quinoline C3) and 20.80 (N–COCH_3_). In the mass spectrum (Fig. 5c) and as shown in mass fragmentation (Fig. 8) Molecular ion peak appeared at [M + , 297; 21.9%)], which losses CH_2_, HCN, H&H_2_O, CN, Cl, and C_2_NH to give fragment peaks at (283; 27.59%), (256; 34%), (237; 31.18%), (211; 21.13%), (176; 39.97%) and (137; 27%) respectively. Other fragment peaks and base peak appears at (295; 18.23%), (280; 23.34%), (264; 19.36), (base peak, 223, 100%), and (188; 26.34%) due to loss of H_2_, CH_3_, O, CN_2_H, and Cl from molecular ion peak.

### Electrochemical experiments

Three electrodes were employed in an electrochemical cell to examine and quantify all electrochemical measurements (PP, EIS, and EFM). The counter electrode was made of graphite, while the working electrode was a sample of CS with a surface area of one cm^2^.

After being cleaned with acetone, rinsed with distilled water, polished with aluminium oxide sheets of varying grades (300–2000), and given a brief opportunity to dry before being submerged in the test solution, the working electrode was put through a series of steps. PP measurements were carried out in accordance with standard working practices utilizing a Gamry Potentiostat Reference 3000-Model number and corrosion software^9,31,32^.

### Surface examination

After being submerged for 24 h in HCl (1.0 M) with and without different concentration of the inhibitors, the surface of CS sheet was studied. The investigated samples were then dried with direct light heat. An energy dispersive X-ray “EDX”-attached scanning electron microscope “SEM” model PrismaE “Thermofisher” was used to characterize the materials' surface morphology and elemental composition. Each sample was fixed on aluminium stubs with standard diameters using a carbon double sticky tape. A 30 kV accelerating voltage was used for the SEM analysis of each sample.

## Result and discussion

### Chemistry

2-chlorobenzo[h]quinoline-3-carboaldehyde (II) is the key structure in our work, which was prepared in previous literature^33–38^ those pathways are illustrated in Scheme 1. An appropriate, N-aryl acetamide such as N-(naphthalen-1-yl) acetamide (I) was synthesised via the acetylation reaction of α-naphthylamine with an acetylating agent such as acetic anhydride. Then, the synthesized (I) was mixed with the Vilsmeier-Haack reagent.

**Figure 9:**
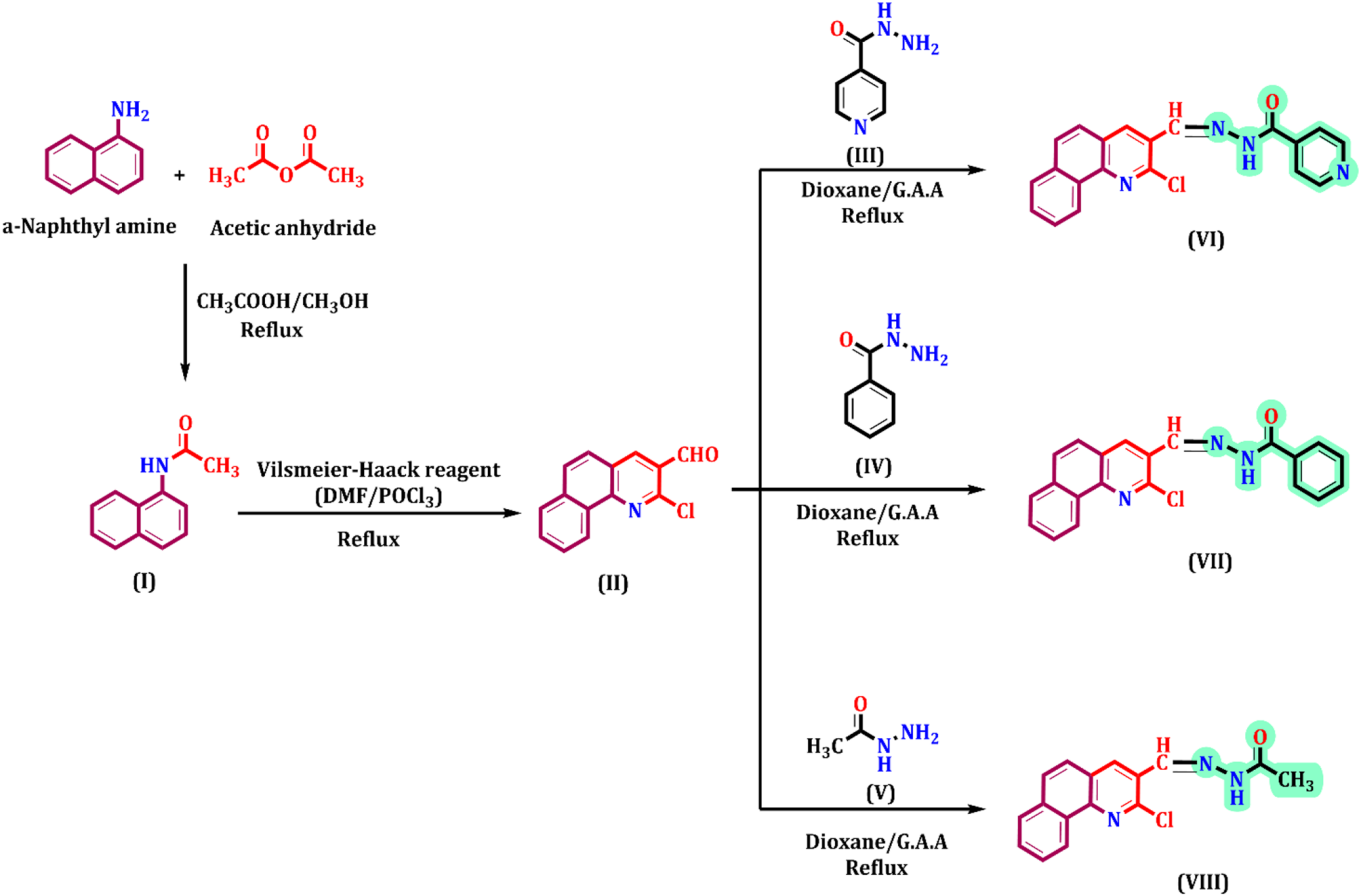
Synthesis of N-(benzo[h]quinoline-3-ylmethylene) hydrazide inhibitors (VI–VIII).

The reactivity of 2-chlorobenzo[h]quinoline-3-carboaldehyde (II) with hydrazide derivatives was investigated. Thus aldehyde (II) reacted with isonicotinoyl hydrazide, benzoyl hydrazide, and acetyl hydrazide (III–V) to afford isonicotinoyl hydrazone, benzoyl hydrazone and acetyl hydrazone derivatives (VI–VIII) of 2-chlorobenzo[h] quinoline-3-carbaldehyde, respectively. This occurs in dioxane/acetic acid under refluxing temperature as shown in Fig. 9. The structure of new corrosion inhibitors was confirmed by their spectral data (See Experimental section).

### Electrochemical studies

#### Open circuit potential (OCP) curves

**Figure 10:**
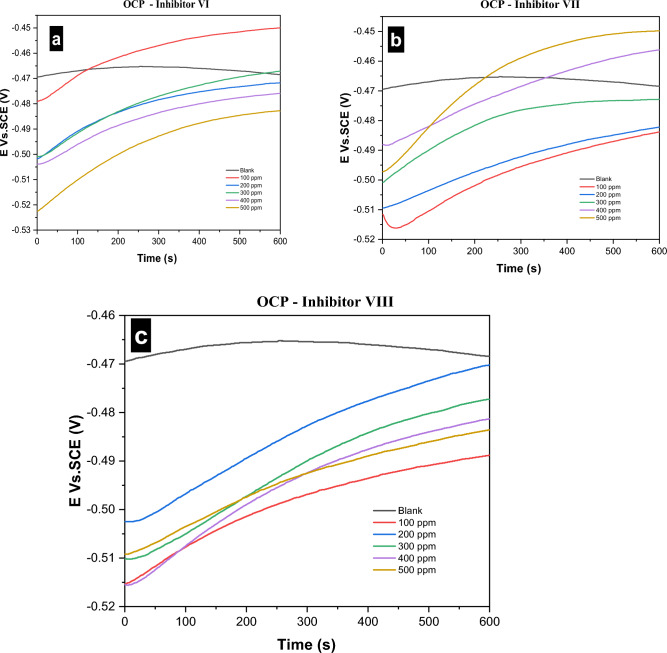
(**a**–**c**) OCP curves for the corrosion of carbon steel in 1.0 M HCl in absence and presence of different concentrations of investigated inhibitors (VI, VII and VIII) at 25 °C.

OCP curves for the corrosion of carbon steel in 1.0 M HCl in absence and presence of different concentrations of investigated inhibitors (VI, VII and VIII) at 25 °C are shown in Fig. 10.

#### Potentiodynamic polarization (PP) measurements

**Figure 11:**
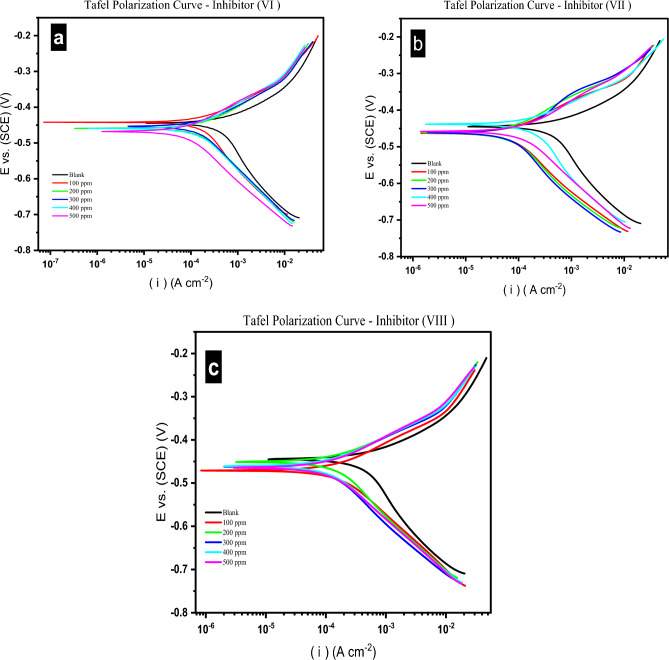
(**a**–**c**) Potentiodynamic polarization curves for the corrosion of carbon steel in 1.0 M HCl in absence and presence of different concentrations of investigated inhibitors (VI, VII, and VIII) at 25 °C.

PP curves are shown in Fig. 11, the CS electrode was immersed in HCl (1.0 M) with and without of various concentrations of the investigated inhibitors (VI, VII and VIII) at 25 °C.

Depending on Tafel plots, the electrochemical parameters were evaluated and mentioned in Table 2, where (βa: Anodic Tafel Slopes, βc: Cathodic Tafel Slope, i_corr_: Current Density, E_corr_ Vs.SCE: Corrosion Potential, K: the Rate of Corrosion, θ: Surface Coverage and η_pp_%: the efficiency of inhibition^39^. In HCl(1.0 M), the effects of all the examined inhibitors on the rates of hydrogen evolution were assessed, and were indicated by changes in the value of βc with raising the concentration of the examined inhibitor. It can be seen from the data in Table 2 that the corrosion rate is in directly proportional relation with the concentrations of investigated inhibitors (VI, VII and VIII), the next equation can be used to calculate the inhibitory effectiveness. (1)^40^.

1 $$\eta_{{{\text{pp}}}} \% = {\uptheta} \times 100 = \left[ {\left( {{\text{\it{i}}}^{0}_{{{\text{\it{corr}}}}} {-}{\text{\it{i}}}_{{{\text{\it{corr}}}}} } \right)/{\text{\it{i}}}^{0}_{{{\text{\it{corr}}}}} } \right] \, \times 100$$

where i^0^_corr_ & i_corr_ : the current densities of the corrosion without and with different doses the investigated inhibitors, respectively.

**Table 2:** Potentiodynamic electrochemical parameters for CS samples immersed in 1.0 M HCl, in absence and presence of different concentration of investigated inhibitors at 25 °C.

Inhibitor #	C (ppm)	βa (mV dec^−1^)	βc (mV dec^−1^)	i_corr_ (uA)	E_corr_ vs.SCE (mV)	K (mpy)	Chi Squared	θ	ηpp%
Blank	–	115.10	271.20	703	− 445	321.4	41.25	–	–
Inhibitor VI	100	95.30	189.00	239	− 442	17.36	29.13	0.66	66.00
	200	100.30	152.30	222	− 459	16.16	18.97	0.68	68.42
	300	94.60	154.00	206	− 454	15.01	23.46	0.71	70.70
	400	98.60	147.80	189	− 459	13.75	11.41	0.73	73.12
	500	88.40	131.50	98.6	− 468	7.174	18.43	0.86	85.97
Inhibitor VII	100	89.20	199.10	222	− 438	16.13	20.59	0.68	68.42
	200	96.20	140.70	145	− 464	10.56	5.537	0.79	79.37
	300	92.20	141.40	131	− 458	9.533	4.19	0.81	81.37
	400	82.10	139.30	80.1	− 463	5.826	21.39	0.89	88.61
	500	83.10	140.30	68	− 462	4.95	9.555	0.90	90.33
Inhibitor VIII	100	99.10	141.30	219	− 471	15.97	17.37	0.69	68.85
	200	94.30	161.10	200	− 451	14.54	29.38	0.72	71.55
	300	91.60	146.30	146	− 462	10.64	26.71	0.79	79.23
	400	78.80	134.10	133	− 461	9.644	4.968	0.81	81.08
	500	76.50	128.80	123	− 464	8.915	0.35	0.83	82.50

Figure 11 further shows that the anodic curves changed by less than 85 mV in all inhibitors doses into the region of negative corrosion potential, indicating that the examined inhibitors fall under the category of mixed-type corrosion inhibitors. In case of the change in the potential > 85 mV, the inhibitors can be considered both cathodic/anodic type inhibitor^31,41^. The inhibition efficiency of (VI, VII and VIII) achieved the maximum values at 85.97, 90.33 and 82.50 at the highest concentration (500 ppm) respectively.

#### Electrochemical frequency modulation

**Figure 12:**
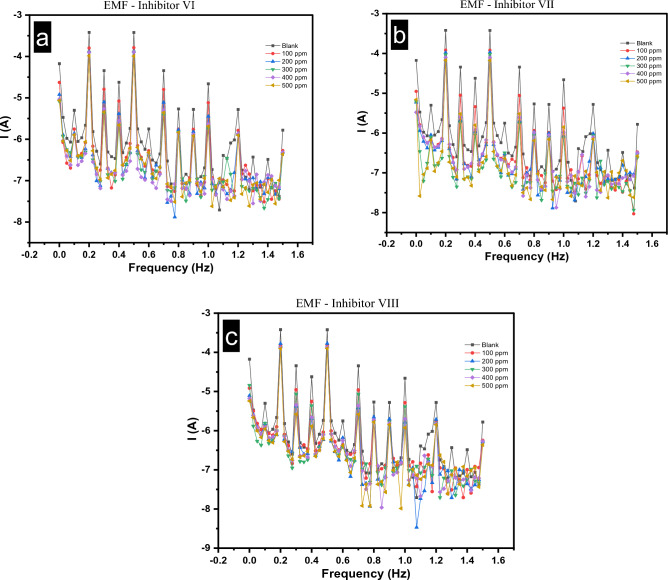
(**a**–**c**) Intermodulation spectrum for carbon steel in 1.0 M HCl in absence and presence of different concentrations of investigated inhibitors (VI, VII and VIII) at 25 °C.

EMF is an intermodulation technique that assesses the current density response at sums, differences, and multiples of the input frequency using a dual frequency potential perturbation^42^. This making it effectively useful for assessing the corrosion rates and the efficacy of corrosion inhibition. It is also regarded as a very characteristic electrochemical technique, since it is a non-destructive, rapid technique, corrosion rate can be calculated without the need to determine the Tafel slopes (βa and βc) and the good strength point is the internal check on the measurements by comparing the obtained results with the theoretical values of the causality factors^43,44^. As shown at EMF obtained graph in Fig. 12, the intermodulation spectra describe the relation between the current responses for different samples in presence of various concentrations from 100 to 500 ppm of investigated inhibitors. All kinetic parameters are shown in Table 3 as the current density (i_corr_), anodic(βa) and cathodic (βc) Tafel constant slopes, corrosion rate (K) and the casualty factors (CF_2_ & CF_3_) as a function of concentration of investigated inhibitors (C, ppm). The following formulae were used to determine the corrosion efficiency (η_EFM_%) and the surface coverage (θ):

2 $$\eta_{{{\text{EFM}}}} \% = \uptheta \times { 1}00 = \left[ {\left( {{\text{i}}^{0}_{{{\text{corr}}}} {-}{\text{ i}}_{{{\text{corr}}}} } \right)/{\text{i}}^{0}_{{{\text{corr}}}} } \right] \times {1}00$$

where i^0^_corr_ & i_corr_ : the current densities in absence and presence of different doses the investigated inhibitors, respectively.

**Table 3:** EMF Parameters for CS samples immersed in 1.0 M HCl, in absence and presence of different concentration of investigated inhibitors at 25 °C.

Inhibitor #	C (ppm)	i_corr_ (uA cm^−2^)	βa (mV dec^−1^)	βc (mV dec^−1^)	K (mpy)	CF (2)	CF (3)	θ	η_EFM_%
Blank	–	734.7	86.01	233.70	335.7	1.993	3.045	–	–
Inhibitor VI	100	341.8	97.06	238.50	156.2	2.011	3.246	0.53	53.48
	200	229.5	94.58	145.20	104.9	2.038	3.303	0.69	68.76
	300	228	104.40	130.70	104.2	2.038	3.075	0.69	68.97
	400	225.2	100.70	135.40	102.9	2.059	2.875	0.69	69.35
	500	166.3	92.48	122.60	75.99	2.088	2.842	0.77	77.36
Inhibitor VII	100	273.6	110.80	221.10	125	2.012	3.18	0.63	62.76
	200	196.8	112.70	129.80	89.92	2.106	3.209	0.73	73.21
	300	178.5	114.80	132.40	81.56	2.145	3.004	0.76	75.70
	400	146.3	98.65	123.50	66.86	2.201	4.244	0.80	80.09
	500	137.4	102.50	127.80	62.76	2.2	3.189	0.81	81.30
Inhibitor VIII	100	286	95.91	166.30	130.7	2.046	3.316	0.61	61.07
	200	281.5	101.10	118.70	128.6	2.169	3.239	0.62	61.69
	300	225	90.90	143.10	102.8	2.04	3.569	0.69	69.38
	400	223.2	98.08	122.70	102	2.121	2.806	0.70	69.62
	500	209.4	102.20	117.60	95.68	2.101	3.282	0.71	71.50

The experimental causality factor's values were relatively close to its theoretical values (2 and 3 respectively), which indicating the good quality and the low error of the obtained results^8^. It is obvious from the results in Fig. 12 and Table 3, that the three investigated inhibitors have a good corrosion inhibition efficiency, where the value of (i^0^_corr_) is higher than (i_corr_) for all investigated inhibitors, and the values of (i_corr_) are inversely correlated with inhibitor concentrations, indicating that when inhibitor concentrations rise, inhibition efficiency rises as well. Also, the corrosion inhibition efficiency of the inhibitor VII seem better than VI & VIII, this is evident when we compared the (i_corr_) value of VII with corresponding values of VI & VIII taking the corresponding concentrations into account.

#### Electrochemical impedance spectroscopy measurements

**Figure 13:**
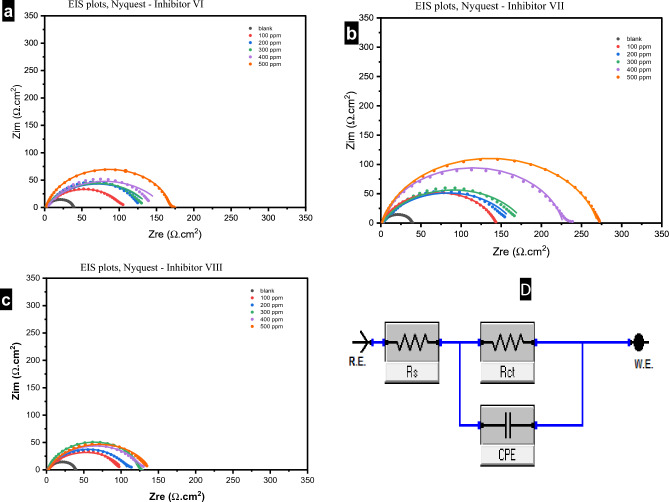
(**a**–**c**) Nyquist plots for carbon steel in 1.0 M HCl in absence and presence of different concentrations of investigated inhibitors (VI, VII and VIII) at 25 °C, and (**D**) comparable circuit for impedance data fitting.

**Figure 14:**
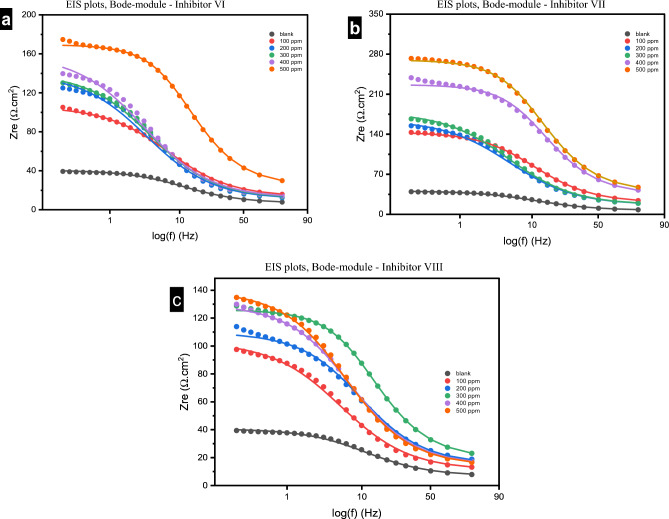
(**a**–**c**) Bode Modulus plots of impedance spectra for steel in 1.0 M HCl in absence and presence of different concentrations of investigated inhibitors (VI, VII and VIII) at 25 °C.

**Figure 15:**
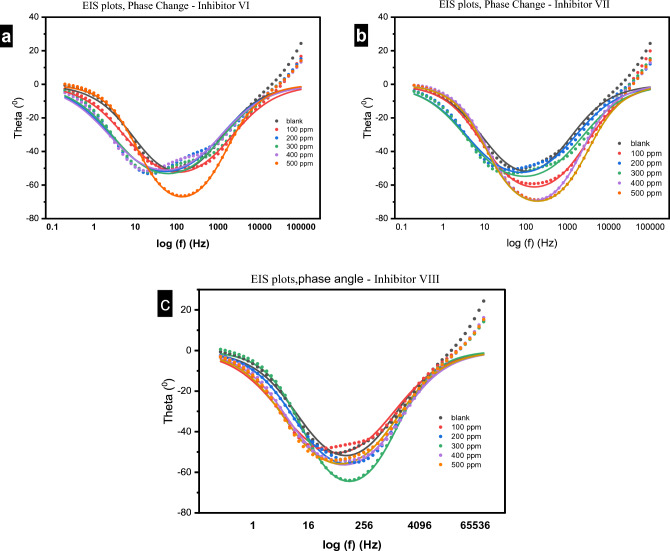
(**a**–**c**) Bode phase change plots of impedance spectra for carbon steel in 1.0 M HCl in absence and presence of different concentrations of investigated inhibitors (VI, VII and VIII) at 25 °C.

The corrosion inhibition efficiency was evaluated for the novel inhibitors (VI, VII, VIII) using the EIS technique, which oriented to the electrochemical corrosion properties^45^. The effect of inhibitors doses on EIS behaviour were investigated after immersing the carbon steel sample into HCl(1.0 M) with and without various concentration of inhibitors from 100 to 500 ppm at ambient temperatures. The results are presented as Nyquist, Bode-module, and Bode-phase angle plots in Figs. 13, 14, and 15, respectively.

As Nyquist plots illustrate, the semicircles shape of the impedance response indicates the charge transfer corrosion mechanism, and the increasing of their size significantly with rising the concentrations of investigated inhibitors indicates enhancing of corrosion resistance, which is mainly caused by an improvement in the protective layer on the metal surface^46^. The continuity appearing semicircle shape in presence of the investigated inhibitors suggests that the mechanism of carbon steel dissolving remained unchanged^47^.

The faradic process interprets the existence of the single capacitive loop, including a single charge transfer resistance and double layer capacitance element^48^. The defect in the shape of the semicircles is acceptable especially in case of using the solid electrodes, this is happened because of the surface heterogeneity of the electrode, inhibitor adsorption, and microscopic roughness^49^. According to the element CPE (constant phase element), there is a double electrical layer capacity at the electrode/electrolyte interphase. By using the CPE parameters (Y_o_ and n) from the following equation, the double-layer capacitance (C_dl_) was determined^50^:

3 $$C_{dl} = \left( {Y_{0} R_{P}^{1 - n} } \right)^{1/n}$$

Table 4 shows that increasing the concentration of inhibitors causes the charge transfer resistance (R_ct_) to increase and the magnitude of the constant phase element (Y_0_) and double layer capacitance (*C*_*dl*_) to decrease. The presence of a passive layer on the electrode surface and the formation of a mass and charge transfer barrier, which successfully inhibit corrosion, are effects that are linearly related to the inhibitor concentration^51^. This happens in the following order: VI < VIII < VII which indicates an increase in the corrosion inhibition efficiency in the same order.

**Table 4:** EIS Parameters for CS samples immersed in 1.0 M HCl, in absence and presence of different concentration of investigated inhibitors at 25 °C.

Inhibitor#	C (ppm)	Rct (Ω.cm^2^)	Rs (Ω.cm^2^)	Y0 (Ω^−1^s^n^cm^−2^)	Cdl (F cm^−2^)	Θ	ηz %
Blank	–	39.5900	1.1470	1.08E−03	4.01E−04	–	–
Inhibitor VI	100	104.7000	1.6180	7.18E−04	2.57E−04	0.62	62.19
	200	138.3000	1.9910	9.90E−04	4.31E−04	0.71	71.37
	300	138.9000	1.8870	8.65E−04	3.75E−04	0.71	71.50
	400	157.700	2.191	9.71E−04	4.22E−04	0.7490	74.90
	500	167.6000	2.1930	1.36E−04	8.24E−05	0.76	76.38
Inhibitor VII	100	142.5000	1.4750	2.79E−04	1.23E−04	0.72	72.22
	200	163.4000	2.4110	6.16E−04	2.45E−04	0.76	75.77
	300	177.5000	1.8430	5.75E−04	2.37E−04	0.78	77.70
	400	224.7000	2.1890	9.37E−05	5.75E−05	0.82	82.38
	500	268.4000	1.9010	8.95E−05	5.28E−05	0.85	85.25
Inhibitor VIII	100	101.5000	2.0540	9.45E−04	3.64E−04	0.61	61.00
	200	107.9000	1.9770	4.42E−04	1.81E−04	0.63	63.31
	300	124.1000	2.0840	1.89E−04	1.09E−04	0.68	68.10
	400	128.4000	1.8480	5.13E−04	2.18E−04	0.69	69.17
	500	137.8000	2.0180	5.52E−04	2.40E−04	0.71	71.27

The efficiency of corrosion (ηz%) and the surface coverage(θ) were calculated from the following equation:

4 $$\eta{{{\text{z}}}} \% = \uptheta \times {1}00 = \left( {{\text{R}}_{{{\text{ct}}}} {-}{\text{R}}_{{{\text{ct}}}}^{`} } \right)/{\text{R}}_{{{\text{ct}}}} \times {1}00$$

where R_ct_ & R`_ct_ : the charge transfer resistance with and without the investigated inhibitors, respectively.

The Bode phase angle diagram reaches a peak in the region of intermediate frequency seen in Fig. 15, demonstrating that there is only one time constant^52^. According to Fig. 14, the impedance modulus increased from 40 to 170, 279 and 135 Ω.cm^2^ for the blank and in presence of the studied inhibitors VI, VII and VIII respectively. This rise in the impedance modulus indicates the good inhibition efficiency of the studied inhibitor^53^.

### Adsorption isotherm

**Figure 16:**
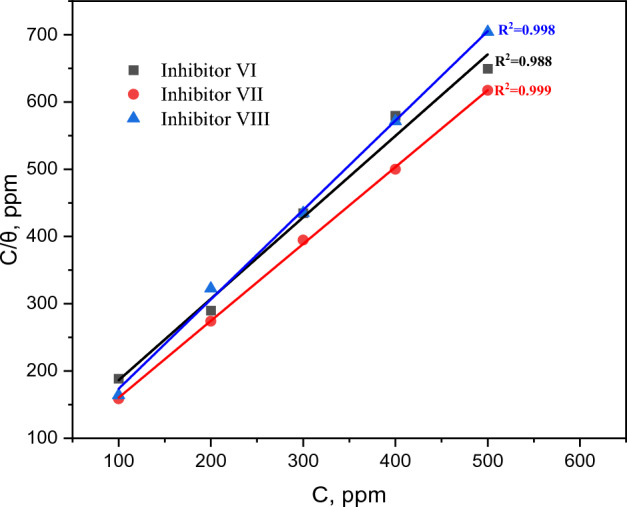
Langmuir adsorption isotherms for investigated inhibitors (VI,VII and VIII) at 25 °C.

Adsorption isotherm can present the mechanism between the examined inhibitors and the surface of carbon steel^54,55^. As shown in Fig. 16, the Langmuir adsorption model (Eq. 5) can correctly describes the adsorption of investigated inhibitors (VI,VII and VIII) on the carbon steel surface^56^.

5 $$\frac{C}{\uptheta } = \frac{1}{K\;ads} + C$$

where C is the concentration of investigated inhibitors, θ is the surface coverage, and K_ads_ is equilibrium constant.

### Surface examination

#### Scanning electron microscope

**Figure 17:**
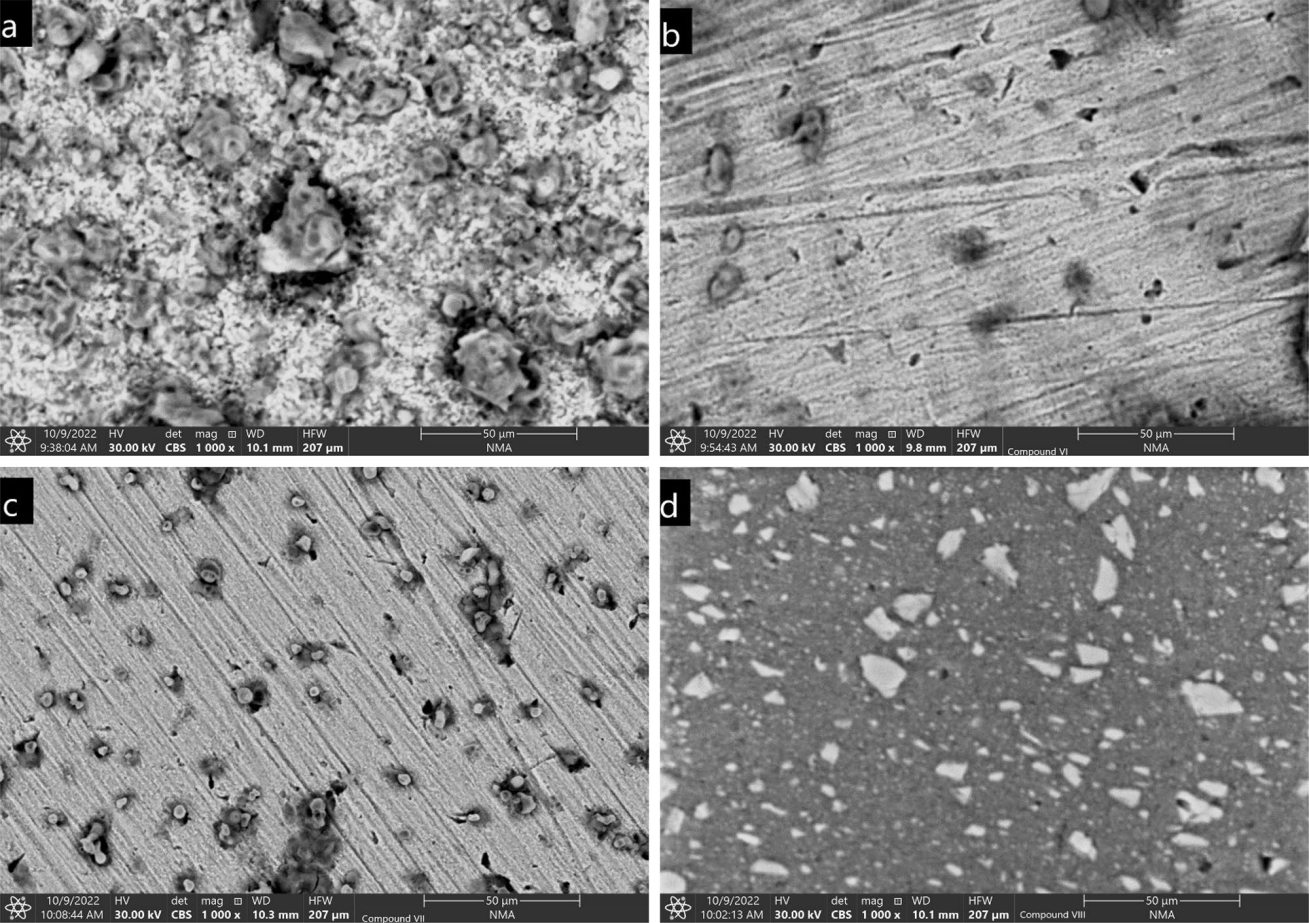
(**a**–**d**) SEM micrographs of steel samples after 24 h immersion 1.0 M HCl (**a**) in absence of inhibitors and (**b**–**d**) in presence of investigated inhibitors (VI, VII and VIII).

Using SEM technique, the morphology of the CS specimen was investigated to compare the surface changes that resulted from immersion it in corrosive medium in the absence of the investigated inhibitors (blank) and in the presence of the investigated compounds (VI, VII and VIII). All CS samples were exposed to HCl (1.0 M) for 24 h at room temperature 25 °C then removed from 1.0 M HCl solution standing one h to be dried and ready for testing. As shown in Fig. 17, (a) represents the micrograph of control sample in the absence of inhibitors the wide corroded areas, apparent deteriorations and decompositions are observed. In comparison, the micrographs of the specimen carbon steel samples in presence of the inhibitors, VI, VII and VIII in (Fig. 17 b–d) respectively, their highly corrosion inhibition efficiency could be concluded from the decreasing of localized corrosion areas, the disappearing of deterioration areas and the fit layers which covered the majority of the surface^57,58^. According to the previous results of electrochemical methods and the current SEM results both align to reveal the good corrosion control of the investigated inhibitors by the formation of a passive film on the carbon steel surface.

#### Energy dispersive X-ray (EDX).

Samples of CS sheets were analysed using the EDX technique with immersion in the aggressive solution, without and with the investigated inhibitors (VI, VII and VIII).

**Figure 18:**
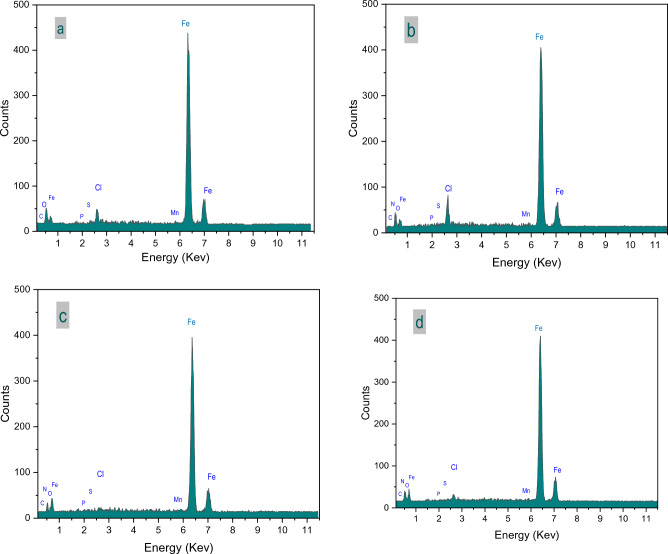
(**a**–**d)** EDX patterns of the film formed on carbon steel surface without immersion (after immersion in HCl; (**a**) in absence of inhibitors and (**b**–**d**) in presence of investigated inhibitors (VI, VII and VIII).

The EDX technique aim is determination of elements that make up the layer on the CS surface, and the intensity of the EDX peaks reveals the concentration of these elements. As shown in the Fig. 18 and Table 5 the presence of the investigated inhibitors in HCl (1.0 M) caused a significant change in the film which formed on the carbon steel sheet. The peaks of Nitrogen which detected in presence of the inhibitors indicate the adoption of inhibitor molecules on the carbon steel surface, which reveals the corrosion inhibition efficiency of the investigated inhibitors.

**Table 5:** EDX Quantitative analysis for carbon steel (CS) surface samples without immersion and after 24 h immersion in 1.0 M HCl in presence and absence of the investigated inhibitors (Compounds VI, VII and VIII).

Element	Baseline (CS Sample)		Control (CS–HCl)		CS–Compound VI		CS–Compound VII		CS–Compound VII	
	Weight %	Atom %	Weight %	Atom %	Weight %	Atom %	Weight %	Atom %	Weight %	Atom %
C	1.23	5.19	3.11	10.41	4.07	13.37	3.38	11.88	1.03	3.79
N	–	–	–	–	2.63	7.41	1.2	3.62	2.52	7.94
O	1.52	4.82	9.97	25.05	5.83	14.37	5.98	15.78	5.12	14.13
Al	1.22	2.29	0.00	0.00	0	0.00	0	0.00	0	0
P	0.37	0.61	0.41	0.53	0.55	0.70	0.48	0.65	0.6	0.86
S	0.26	0.41	0.74	0.93	0.43	0.53	0.59	0.78	0.61	0.84
Cl	0.00	0.00	3.22	3.65	6.2	6.90	1.15	1.37	2.64	3.29
Mn	1.31	1.21	0.95	0.70	1.22	0.88	0.95	0.73	0.87	0.7
Fe	94.09	85.47	81.60	58.74	79.07	55.85	86.27	65.20	86.61	68.46

### Corrosion inhibition mechanism

The electrochemical corrosion data (PP, EMF, and EIS) which obtained in both the absence and presence of investigated inhibitors:

- The reduction in corrosion rate as the examined inhibitors dose increases, indicating the inhibition efficiency depends on concentration.
- The efficiency of inhibition depends on the presence of active centres for adsorption in the molecule and their charge density.

The efficiency of inhibition of the investigated compounds in the corrosive solution was decreased in the following order: Compound VII > compound VI > compound VIII.

Numerous factors, such as charge density, the adsorptive active centres, the molecular size, the mode of adsorption, and the degree to which metal complexes are formed, affect how effectively organic molecules in hydrochloric acid and CS solution inhibit corrosion^59^. Through its active centres, which are heteroatoms with unshared electron pairs or aromatic rings with π-bonding, adsorption can take place.

According to the electrochemical data, compound VIII has the lower protection efficiency, which could be attributable to its less number of active sites and charge density when compared to the other compounds. Further, Compound VII has the best performance then Compound VI, even though both have the same number of active sites and Compound VI has more electronegative heteroatom, according to earlier research, compounds with more electronegative heteroatoms in quinoline derivatives exhibit lower levels of protection than those with fewer electronegative atoms^60^. This could imply that the lone pair of electrons, located on nitrogen inside the branched pyridinyl ring, in this position does not participate directly in adsorption on the CS surface, due to its molecular geometry in space. Further, the phenyl ring has a p-electron density when compared to the pyridinyl group due to the withdrawing effect of Nitrogen in pyridinyl.

## Conclusion

In conclusion, our study successfully prepared and evaluated three new organic compounds based on benzh]quinoline hydrazone derivative as potential corrosion inhibitors. The chemical structures were confirmed using FTIR, Mass, ^1^H-NMR, and ^13^C-NMR techniques. Evaluation of the performance of corrosion inhibition was investigated for carbon steel in HCl (1.0 M) medium. The used electrochemical methods including polarization measurements, electrochemical frequency modulation method (EFM) and electrochemical impedance spectroscopy (EIS). PP and EIS results revealed that the three compounds described as mixed-type inhibitors. Additionally, all electrochemical data showed that compound VII, in all studied concentrations from 100 to 500 ppm, exhibited better corrosion inhibition performance than compounds VI and VIII. According to the results of PP, EMF, and EIS, compound VII's maximum corrosion inhibition efficiency at 500 ppm was 90.33, 82.50, and 81.30, respectively. The formation of a resistive layer on the metal surface following treatment with the inhibitors was confirmed by surface examination using SEM and EDX.

## Data Availability

The datasets used and/or analysed during the current study available from the corresponding author on reasonable request.
